# Deficient repair response of IPF fibroblasts in a co-culture model of epithelial injury and repair

**DOI:** 10.1186/1755-1536-7-7

**Published:** 2014-04-29

**Authors:** Sony Prasad, Cory M Hogaboam, Gabor Jarai

**Affiliations:** 1 Novartis Institutes of Biomedical Research, Respiratory Disease Area, Wimblehurst Road, Horsham, West Sussex RH12 5AB, UK; 2 Department of Medicine, Cedars Sinai Medical Center, AHSP Room A9108, 127 S. San Vicente Blvd, Los Angeles, CA 90048-3311, USA

**Keywords:** Idiopathic pulmonary fibrosis, IPF, Co-culture, Wound healing, PDGFR, bFGF, HGF, PDGF

## Abstract

**Background:**

Idiopathic pulmonary fibrosis (IPF) is a progressive disorder marked by relentless fibrosis and damage of the lung architecture. A growing body of evidence now suggests that IPF progresses as a result of aberrant epithelial-fibroblast crosstalk. Injured epithelia are a major source of growth factors such as PDGF which guide resident fibroblasts to injury sites.

**Results:**

In this study, we utilized a novel co-culture system to investigate the effect of fibroblast phenotype on their response to epithelial injury. Fibroblasts from normal lungs (NHLF) responded to epithelial injury and populated the wound site forming a fibroblast plug/mechanical barrier which prevented epithelial wound closure. IPF fibroblasts were impaired in their response to epithelial injury. They also expressed reduced PDGFRα compared to NHLFs and were defective towards PDGF-AA mediated directional movement. Neutralization of PDGF-AA and pan-PDGF but not PDGF-BB reduced the injury response of NHLFs thereby preventing the formation of the mechanical barrier and promoting epithelial wound closure. Co-culture of epithelial cells with IPF fibroblasts led to marked increase in the levels of pro-fibrotic growth factors - bFGF and PDGF and significant depletion of anti-fibrotic HGF in the culture medium. Furthermore, IPF fibroblasts but not NHLFs induced a transient increase in mesenchymal marker expression in the wound lining epithelial cells. This was accompanied by increased migration and faster wound closure in co-cultures with IPF fibroblasts.

**Conclusions:**

Our data demonstrate that the IPF fibroblasts have an aberrant repair response to epithelial injury.

## Background

Idiopathic pulmonary fibrosis (IPF) is a chronic, progressive lung disease of poorly defined etiology and pathogenesis. Decline in lung function eventually results in respiratory failure and death within 3 to 5 years of diagnosis. Usual interstitial pneumonia (UIP), the histological presentation of IPF, is marked by patches of fibrotic tissue in areas of normal appearing lung [1]. Presence of fibroblastic foci underneath damaged-reparative epithelium and basement membrane is a hallmark of the disease [2]. Disease progression is accompanied by matrix deposition, extensive scarring, and alveolar destruction leading to the characteristic honeycomb lesions seen in IPF [3].

Despite extensive research in understanding disease pathogenesis, IPF remains poorly defined. The hypothesis that is currently most widely accepted proposes that IPF is initiated by repeated micro-injuries to the alveolar epithelium that lead to aberrant and irreversible scarring.

During normal repair the injured alveolar epithelium secretes growth factors, cytokines, and chemokines that are essential for epithelial cell migration and differentiation. They also act as chemo-attractants and mitogens for fibroblasts that migrate into the wound site [4] and are critical for generating connective tissue needed for the subsequent re-epithelialization, secretion of growth factors and cytokines, and wound contraction [5,6]. Fibroblasts are then removed from the wound site primarily by apoptosis [7]. Wound repair is a highly regulated response and aberrant wound healing and persistent presence and activation of fibroblasts can result in pathological scarring [8].

Several studies have reported differences between fibroblasts derived from healthy lung and IPF lung [9]. Altered production of HGF [10], MCP-1 [11] and prostanoids [10,12], increased IL-6 mediated proliferation [13], and reduced FasL mediated apoptosis [7] have been reported in IPF fibroblasts. There is also evidence for a synthetic phenotype for IPF fibroblasts with increased secretion of pro-fibrotic mediators and extracellular matrix, lower growth rate, and increased apoptosis [9]. It has been suggested that these apparent differences may reflect the heterogeneous nature of the fibroblasts population.

Cell-cell contact between epithelial cells and fibroblasts appears to be important in signaling cascades and critical for wound repair [14]. In order to investigate epithelial-fibroblast crosstalk in the development of IPF, we sought to examine the effect of fibroblast phenotypes on epithelial repair using an epithelial scratch wound model in a unique co-culture system. Using this model system we demonstrate that in response to epithelial injury, NHLFs and IPF fibroblasts stimulate a differential epithelial repair response. Furthermore we investigate the mechanism driving these phenotypic differences and identified a role for platelet-derived growth factor (PDGF), hepatocyte growth factor (HGF), and basic fibroblast growth factor (bFGF) expression and signaling. We also show that IPF fibroblasts promote mesenchymal marker expression in the reparative epithelial cells.

## Results

### The effect of fibroblast phenotype on scratch wound closure

**Figure 1:**
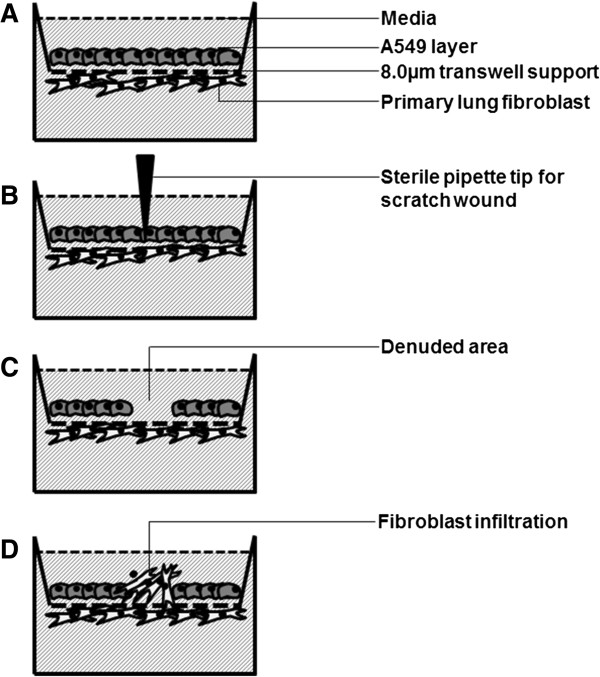
**Schematic diagram depicting epithelial-primary fibroblast co-culture scratch wound model. (A)** A549 and fibroblasts are attached on either side of trans-well insert coated with collagen. **(B)** Scratch wound is created by running a sterile pipette tip across. **(C)** Resultant denuded area. **(D)** Fibroblast transmigration across trans-well into denuded area.

**Figure 2:**
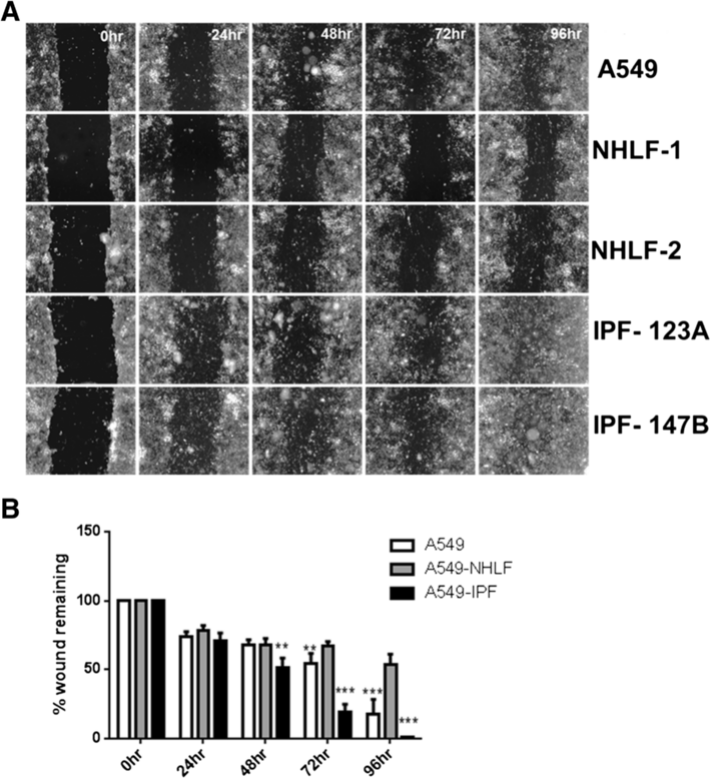
**Effect of fibroblast phenotypes on re-epithelialization of A549 scratch wounds. (A)** Representative real time images illustrating the effect of co-culture with lung fibroblast from two normal and two IPF donors on re-epithelialization of A549 (epithelial) scratch wound. Co-culture with NHLFs prevented while presence of IPF fibroblasts promoted wound closure compared to epithelial mono-culture. **(B)** Compared to A549 mono-cultures, wound closure was significantly higher with IPF fibroblasts at the 72 h and 96 h time points closure whereas it was significantly inhibited by NHLFs. Furthermore, compared to IPF co-cultures, there was significantly less wound closure in NHLF co-cultures at 48 h, 72 h, and 96 h. Results are representative of n = 3 experiments for each donor. * *P* <0.05, ** *P* <0.01, ***P <0.001 compared with A549-NHLF.

The extent of re-epithelialization was investigated by wound closure of PKH26-labeled A549 cells in mono-cultures or in co-cultures with NHLFs or IPF fibroblasts over a 96-h time-course (Figure 1). Epithelial wound closure was significantly enhanced in the presence of IPF fibroblasts by 48 h compared to NHLF co-cultures and by 72 h compared to A549 mono-cultures. This difference increased further by 72 h and 96 h. Compared to A549 mono-cultures, co-culture with NHLFs significantly inhibited wound re-epithelialization by A549 cells at 72 h and 96 h post injury. These results were consistent when using multiple NHLF and IPF donors (Figure 2A, B).

### Differential recruitment of normal and IPF fibroblasts into epithelial wounds

**Figure 3:**
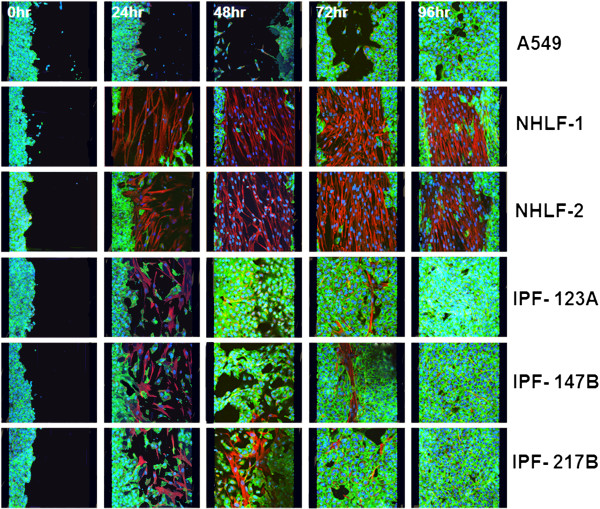
**Immunofluorescence staining showing fibroblast invasion into epithelial scratch wounds.** Representative images showing that sub-epithelial normal fibroblasts (NHLF-1 and NHLF-2) transmigrated into the denuded area and filled the wound progressively forming a mechanical barrier preventing re-epithelialization. Fewer IPF fibroblasts (IPF-123A, 147B, and 217B) transmigrated and promoted A549 migration and re-epithelialization. Green: e-cadherin, Red: vimentin, Blue: DAPI.

**Figure 4:**
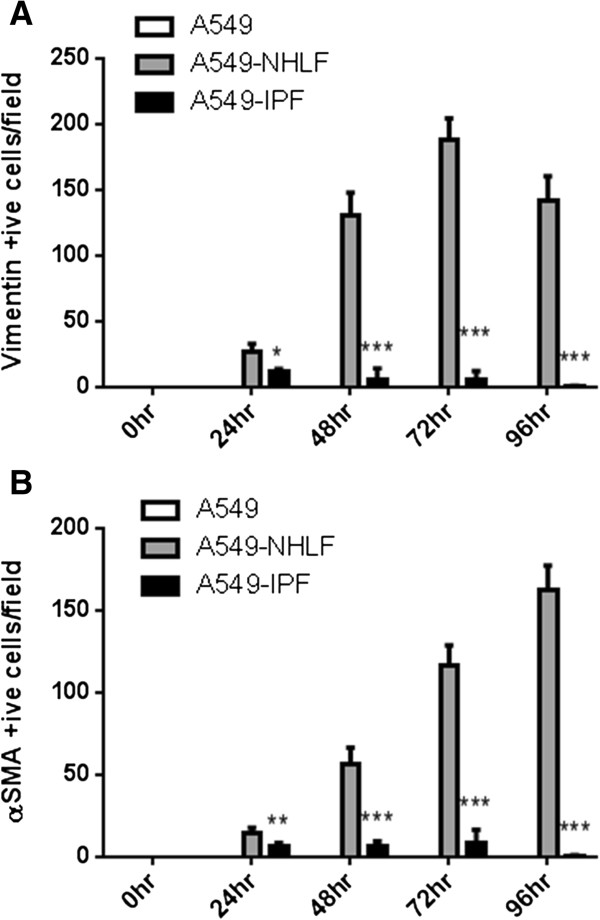
**Recruitment of normal and IPF fibroblasts into epithelial scratch wounds.** The number of NHLFs or IPF fibroblasts invading epithelial wounds was quantified per HPF by employing immunofluorescent staining. A549 cells were identified as e-cadherin/DAPI positive cells; fibroblasts were identified as vimentin/DAPI or α-SMA/DAPI positive cells. **(A)** The number of vimentin/DAPI positive fibroblasts from normal donors (NHLF) populating into the A549 scratch wound was significantly greater than those from donors suffering with IPF (IPF) in comparable culture conditions. **(B)** This was further demonstrated by α-SMA/DAPI positive fibroblasts in separate experiments and showed similar trends as seen in (A). *P <0.05, **P <0.01, ***P <0.001 compared with A549-NHLF.

Next we investigated how epithelial cells and fibroblasts contributed to epithelial repair. NHLFs and IPF fibroblasts responded differentially to epithelial wounding. While NHLFs sensed and migrated in to wound area progressively in response to epithelial injury, IPF fibroblasts appeared to be characterized by a reduced migratory phenotype. By 48 to 96 h, the entire wound area was populated with NHLFs in NHLF-A549 co-cultures leading to the formation of a fibroblast plug. The number of NHLFs populating the epithelial wound area remained significantly higher compared to IPF fibroblast-A549 co-cultures between 48 and 96 h. IPF fibroblasts failed to populate the scratch wound area and these co-cultures exhibited complete re-epithelialization of the wound by the A549 cells by 72 to 96 h (Figures 3 and 4A; Figure 4B; Additional file 1). Presence of sub-epithelial fibroblasts and their phenotypes also had a marked effect on the rate of epithelial wound closure. NHLFs prevented while IPF fibroblasts promoted the rate of wound closure compared to A549 mono-cultures (Figure 2A). These differences were significant from 48 h for IPF co-cultures and at the 72 h time point for NHLF co-cultures (Figure 2B).

### Altered growth factor profile in conditioned medium in IPF fibroblast co-cultures compared to normal fibroblasts

**Figure 5:**
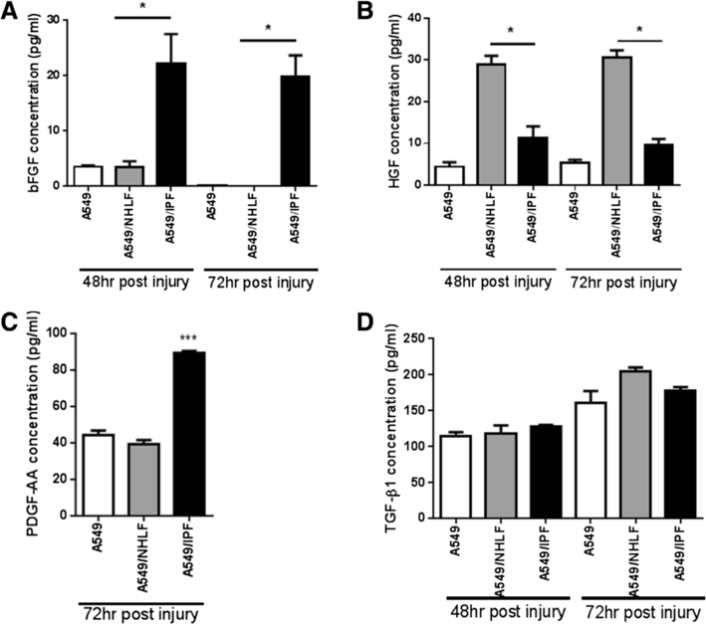
**The effect of fibroblast phenotype on growth factor profile in culture medium. (A)** bFGF concentration in conditioned medium 48 h or 72 h after scratch wound. **(B)** HGF concentration 48 h or 72 h post wounding. **(C)** PDGF-AA concentration 72 h post wounding. **(D)** total TGF-β1 concentration 48 h and 72 h post scratch wound. Results are representative of triplicate experiments, each with multiple donors. * *P* <0.05, ***P <0.001 compared with A549-NHLF.

Cell-cell communication via direct contact or through soluble factors may explain the differential behavior we observe. To investigate if this was the case, the levels of secreted growth factors were analyzed. There was no significant difference in the level of bFGF, HGF, transforming growth factor beta-1 (TGF-β1), and platelet-derived growth factor-AA (PDGF-AA) in conditioned medium (CM) from either NHLF or IPF fibroblast mono-cultures (Additional file 2). In contrast, in co-culture experiments we detected significant differences. In CM from IPF co-cultures, the level of bFGF was significantly elevated (five- to seven-fold) at 48 h compared to A549 alone and NHLF co-cultures. This difference was even more pronounced at 72 h with a nearly 20-fold increase in bFGF in IPF co-cultures (Figure 5A). There was also a two-fold increase in the level of PDGF-AA in IPF co-cultures compared to A549 mono-cultures or NHLF co-cultures at 72 h (Figure 5C). On the other hand, the level of HGF was significantly higher in NHLF co-cultures at both time points compared to IPF co-cultures and to A549 mono-cultures (Figure 5B). There was no difference in the level of total TGF-β1 in CM among the different culture conditions (Figure 5D). In the absence of A549, the level of TGF-β1 was extremely low (Additional file 2), suggesting that A549 is the major source of this growth factor. The levels of epidermal growth factor (EGF), insulin like growth factor 1 (IGF-1), and platelet-derived growth factor-BB (PDGF-BB) were below detection threshold.

### Effect of the fibroblast phenotypes on mesenchymal marker expression in A549 cells after scratch wound

**Figure 6:**
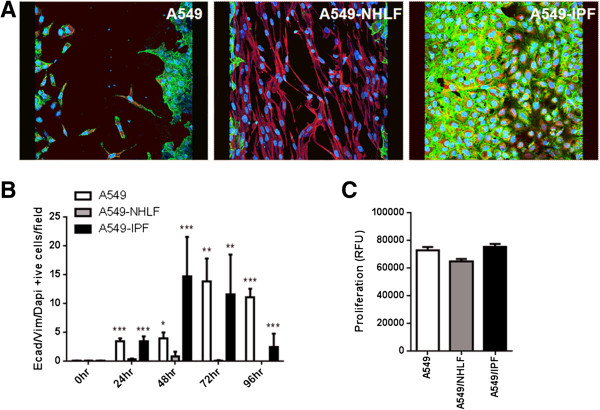
**The effect of fibroblast phenotype on A549 mesenchymal marker expression and proliferation. (A)** Immunofluorescent staining of A549 monoculture, A549-NHLF, and A549-IPF co-cultures on epithelial side showing co-staining e-cadherin/vimentin/DAPI in mesenchymal marker expressing A549 cells at 48 h. **(B)** A549 cells expressing e-cadherin/vimentin/DAPI was significantly greater in mono-cultures and in IPF co-cultures compared to NHLF co-culture. Additionally, at the 48-h time point, mesenchymal marker expression was significantly greater in IPF co-cultures compared to A549 mono-cultures. However, by 72 h, there were significantly fewer epithelial cells expressing vimentin in IPF co-cultures compared to A549 mono-cultures (not shown on graph). **(C)** Conditioned medium at 72-h time point was collected and its effect on A549 proliferation was assessed using a DELFIA assay. There was no difference in the induction of proliferation. *P <0.05, **P <0.01, ***P <0.001 compared with A549-NHLF.

We investigated the effect of epithelial-fibroblast crosstalk on vimentin expression in the repairing A549 cells. A previous study has demonstrated that increased vimentin expression in A549 cells can enhance migration and repair *in vitro*[15]. There were significantly fewer e-cadherin and vimentin co-expressing A549 cells in NHLF co-cultures compared to A549 mono-cultures or in co-cultures with IPF fibroblasts (Figure 6A, B). This difference was already striking at 24 h even before the formation of the ‘fibroblast plug’ as seen in the case of NHLF co-cultures (Figure 3). Compared to A549 mono-cultures, the number of vimentin expressing A549 cells peaked earlier in IPF co-cultures. This was significantly greater at 48 h post scratch, reducing gradually thereafter. By 96 h, when re-epithelialization was complete, there were few or no vimentin co-expressing A549 cells in the IPF co-cultures (Figure 6B).

### Effect of conditioned medium on A549 proliferation

Both epithelial migration and proliferation can contribute to re-epithelialization [16]. We therefore sought to establish if A549 cell proliferation mediated by secreted factors in the CM was an underlying mechanism driving the differences we observed in the response of A549 cells to injury. There was no difference in A549 cell proliferation between cells treated with CM from either epithelial cell mono-cultures or co-cultures with NHLFs or IPF fibroblasts (Figure 6C).

### Receptor expression and PDGF-AA and bFGF mediated migration and proliferation in NHLFs and IPF fibroblasts

**Figure 7:**
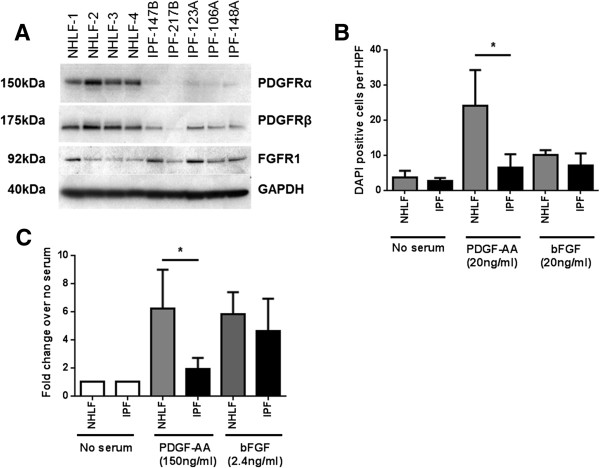
**PDGF receptor expression and PDGF-AA mediated recruitment of NHLF and IPF fibroblasts. (A)** PDGFRα, PDGFRβ, and FGFR1 expression in fibroblasts with GAPDH as loading control. PDGFRα expression was markedly reduced in all IPF donors (n = 5) studied compared to the NHLF donors (n = 4). The expression of PDGFRβ expression was reduced in IPF fibroblasts and exhibited donor to donor variability. FGFR1 expression was variable across all donors irrespective of phenotype. **(B)** IPF fibroblasts showed significantly impaired migratory response to PDGF-AA whereas no difference was seen under baseline conditions. There was no difference in bFGF-mediated migration between phenotypes with both NHLFs and IPF fibroblasts showing significant induction of migration. **(C)** Similarly, significantly higher induction of proliferation was seen in NHLFs in response to PDGF-AA with no difference in bFGF induced proliferation compared to IPF fibroblasts. A concentration of 2× ED50 was used in all assays for each cytokine. *P <0.05.

To investigate if differential receptor expression contributed to the altered response of IPF fibroblasts to epithelial injury, PDGFRα, PDGFRβ, and FGFR1 expression was analyzed in the two fibroblast phenotypes. PDGFRα expression was consistently reduced in all IPF fibroblasts compared to NHLF donors (Figure 7A). PDGFRβ expression was reduced to a lesser extent and presented a more heterogeneous expression across the IPF donors. The expression of FGFR1 was largely similar between IPF fibroblasts and NHLFs with a slight trend towards increased expression in IPF fibroblasts (Figure 7A).

Fibroblasts can populate epithelial wounds through migration and also proliferation of migrated fibroblasts [17]. We therefore investigated if receptor expression impacted the migratory potential of IPF fibroblasts and NHLFs. We did not see an effect of fibroblast phenotypes on baseline migration in serum-free medium. Furthermore, in the absence of stimuli, the number of fibroblasts migrating through the membrane remained extremely low. PDGF-AA strongly induced a directional migration in both normal and IPF fibroblasts; however, the extent of this response was significantly lower in the case of IPF fibroblasts (Figure 7B) suggesting a key role for PDGFRα in this response. bFGF significantly induced migration of both fibroblast phenotypes compared to baseline. However, no significant difference was seen in bFGF-induced migration between NHLFs and IPF fibroblasts. It is well documented that both PDGF-AA and bFGF can act as chemo-attractants as well as mitogens for fibroblasts [18,19]. In line with this, NHLFs stimulated with PDGF-AA demonstrated six-fold increase in proliferation in our study. Consistent with the reduced PDGFRα levels, IPF fibroblasts were less responsive to PDGF-AA-mediated proliferation with only two-fold induction. Both NHLFs and IPF fibroblasts were equally responsive to bFGF-mediated proliferation with a five- to six-fold induction (Figure 7C).

### Effect of PDGF neutralization on fibroblast transmigration and wound closure

**Figure 8:**
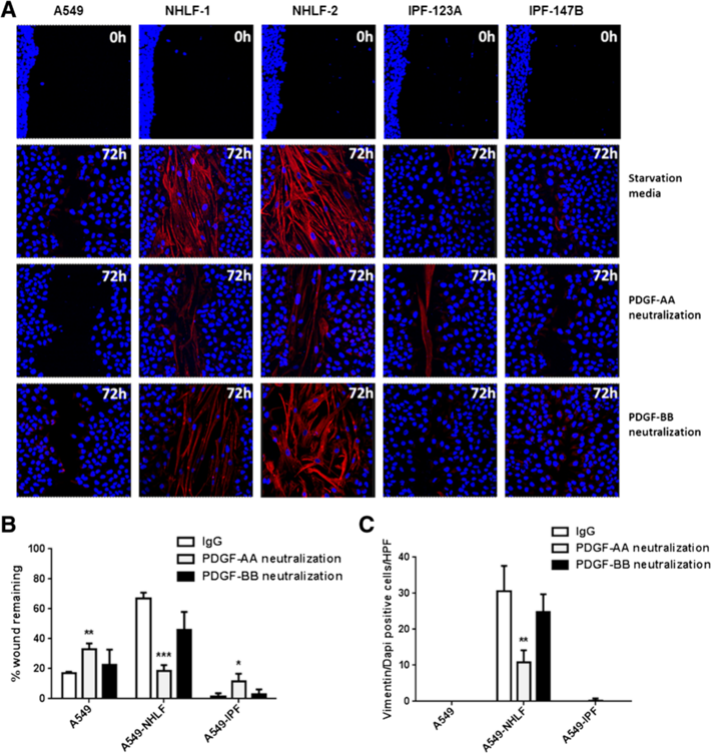
**Effect of PDGF neutralization on epithelial wound closure and fibroblast invasion. (A)** Immunofluorescence staining of the epithelial side of trans-wells (Red: vimentin, Blue: DAPI). **(B, C)** PDGF-AA neutralization resulted in a significant reduction in NHLFs invasion and significant increase in wound closure. There was no effect of PDGF-AA neutralization on IPF fibroblasts; however, in both A549 only controls and IPF co-cultures it significantly reduced wound closure. No significant changes in either fibroblast recruitment or wound closure were observed with PDGF-BB neutralization in any of the cultures. *P <0.05, **P <0.01 compared to IgG control.

To further ascertain if PDGF-driven fibroblast recruitment is a key mechanism driving the differential wound repair response, we assessed the effect of PDGF neutralization on fibroblast migration at 72 h post injury (Figure 8, Additional file 3). Neutralization of PDGF isoforms did not have an effect on IPF fibroblast migration which remained mostly undetectable. On the contrary, PDGF-AA neutralization resulted in a significant reduction in NHLF invasion. There was a three-fold reduction in the number of NHLFs populating the epithelial scratch wound. Recruited fibroblasts occupied a smaller area and also appeared less dense compared to the IgG treated control. PDGF-BB neutralization did not significantly affect migration of NHLFs (Figure 8A, 8C). Neutralization of PDGF-AA significantly inhibited epithelial wound closure in A549 mono-cultures and IPF co-cultures compared to IgG treated controls. Consistent with reduced NHLF invasion the effect of PDGF-AA was reversed in the case of NHLF co-cultures, where the PDGF-AA neutralization led to an increase in epithelial wound closure as the NHLFs failed to fill the epithelial wound area. No significant effect of PDGF-BB neutralization was seen on epithelial closure in any of the three cultures (Figure 8A, 8B). Neutralization using pan-PDGF antibody had a similar effect as PDGF-AA neutralization alone (Additional file 3). These data suggest that PDGF signaling impacts wound repair in our model system by acting both on fibroblast recruitment and on epithelial migration/proliferation and that this effect is primarily driven by the PDGF-AA isoform.

## Discussion

Much emphasis on epithelial-fibroblast crosstalk in IPF has led to a shift in the paradigm that IPF is predominantly an inflammatory disorder to a disorder of alveolar injury and aberrant repair [14]. In IPF, interstitial inflammation, while undoubtedly present, often appears minimal hence it has been proposed that IPF primarily develops via an epithelial/fibroblast route [14]. Indeed, there is evidence that injury of the alveolar epithelium and aberrant repair itself can skew epithelial-fibroblast crosstalk leading to the development of fibrosis [20-22].

In this study, we have used a novel co-culture model of A549 alveolar epithelial cells with a sub-epithelial layer of normal or IPF lung fibroblasts separated by a porous membrane and collagen and demonstrated that fibroblast phenotype impacts epithelial growth factor release and wound healing. This system may prove useful for the future study of mechanisms involved in lung injury and repair in the context of epithelial-fibroblast crosstalk that appears to be a key mechanism in wound healing and fibrosis. The use of A549 cells rather than primary alveolar type II (ATII) cells is an obvious limitation of this model. However, given the difficulties in procuring and maintaining primary human ATII cells in their physiological state *ex vivo*, this system provides a means to study the behavior of cell types from normal or disease lungs in an epithelial repair model.

Fibroblasts and injured alveolar epithelium can be a rich source of growth factors [4]. Interaction of epithelial cells with fibroblasts in an injury-repair setting induces factors needed for epithelial regeneration or in the case of aberrant repair the balance is tipped towards a pro-fibrotic environment [21]. A number of pro-fibrotic growth factors are increased in IPF lungs including TGF-β1, PDGF isoforms, and bFGF [4,23,24] with concurrent decrease in anti-fibrotic factors such as HGF [25]. Consistent with this, we found marked increases in pro-fibrotic growth factors bFGF and PDGF-AA and a significant decrease in HGF in IPF-fibroblast co-cultures. This provides evidence that interaction with disease-derived fibroblasts alone could result in the establishment of a pro-fibrotic environment. Although we did not see a difference in the level of total TGF-β1, we cannot rule out the possibility of increased local activation of latent TGF-β1 initiated by epithelial-fibroblast contacts [26].

Injured alveolar epithelium can be a major source of PDGF isoforms [27] and our results substantiate these findings. The level of secreted PDGF-AA in repairing A549 mono-cultures was similar to NHLF co-cultures. IPF co-cultures, on the other hand, exhibited a marked increase in the level of PDGF-AA, an important fibroblast chemo-attractant and mitogen. It initiates directional movement of fibroblasts into the injury site and promotes their proliferation [19]. Consistent with this, immune-fluorescent staining revealed that normal fibroblasts were progressively recruited into denuded epithelial regions leading to the formation of a fibroblast plug, similar to what has been reported for a mixed co-culture of keratinocytes and fibroblasts [16]. IPF fibroblasts, on the contrary, presented reduced recruitment into epithelial wounds. As fibroblast function is essential for the synthesis of extracellular matrix that is necessary to repair the damaged basement membrane [28,29] and for wound contraction our findings suggest that the compromised response of IPF fibroblasts to epithelial injury may in turn lead to aberrant epithelial repair.

The directional transmigration of NHLFs may be explained by the PDGF-AA gradient created by the injured epithelium. Despite the increased PDGF-AA concentration in IPF co-culture, IPF fibroblasts failed to populate epithelial wounds. In line with this, PDGFRα expression was found to be significantly reduced in all IPF fibroblasts. IPF fibroblasts also presented decreased proliferation to PDGF-AA compared to NHLFs. PDGF-AA only signals via the PDGFRα [30] and decrease in its receptor expression, as in the case of IPF fibroblasts, will impair the response to PDGF-AA and hence epithelial repair. A similar unresponsive phenotype was reported in fibroblasts from chronic non-healing skin wounds compared to healthy wounds [31,32]. An unresponsive phenotype could result in a compensatory increase in PDGF-AA as is seen in case of IPF co-culture similar to what has been described in non-healing wounds [33]. Although, the mechanism for downregulation of PDGFRα is beyond the scope of this study, epigenetic silencing through DNA methylation and histone acetylation could play a role. In IPF, such epigenetic changes have been widely reported [34-38].

bFGF-mediated migration and proliferation and FGFR1 expression were not different between the two fibroblast phenotypes. However, as we found that the concentration of bFGF required to drive fibroblast migration was much higher compared to that needed to induce proliferation, it is possible that at physiological levels increased bFGF in IPF co-cultures could induce proliferation of sub-epithelial IPF fibroblasts without necessarily inducing their transmigration into epithelial wounds.

**Figure 9:**
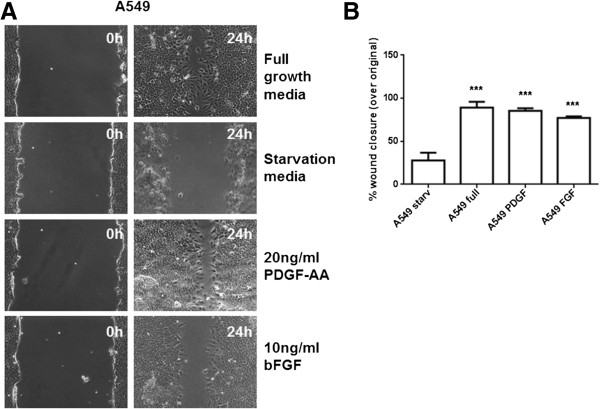
**Effect of rh PDGF-AA and bFGF on A549 wound closure. (A)** Real-time image illustrating wound closure of A549 epithelial monolayers in the presence of different ligands. **(B)** Presence of PDGF-AA and bFGF in culture medium significantly enhanced wound closure to levels comparable to complete medium control. ***P <0.001 compared to A549 under starvation conditions (A549 starv).

Neutralization of PDGF-AA was sufficient to inhibit the recruitment of NHLFs into epithelial wounds. As IPF fibroblasts already show reduced PDGFRα level and subsequent reduced responsiveness to PDGF-AA, further neutralization of PDGF-AA did not impact their response to epithelial injury. In both A549 mono-cultures and IPF co-cultures PDGF-AA neutralization inhibited while in NHLF co-cultures promoted epithelial wound repair. The latter could be the result of reduced migration of normal fibroblasts preventing the formation of the mechanical fibroblast barrier. However, the former finding suggests that A549 might themselves be responsive to PDGF-AA. Indeed, a previous study has shown presence of PDGFRα on the adenocarcinoma A549 cells, that express receptors which are not normally expressed by alveolar epithelial cells [39]. We also found a robust wound closure response in A549 cells driven by PDGF-AA and bFGF (Figure 9). PDGF-BB neutralization did not significantly impact fibroblast invasion or epithelial wound closure. When all PDGF isoforms were inhibited using a pan-PDGF neutralizing antibody, the results were comparable to PDGF-AA neutralization alone and we did not observe an additive effect. This suggests that PDGF-AA is the major isoform affecting fibroblast recruitment as well as wound closure in our model. The reduced recruitment of fibroblasts into the injured area and increased PDGF-AA and bFGF levels in the IPF co-culture are likely to have contributed to enhanced re-epithelialization.

Epithelial-fibroblast crosstalk can lead to changes in secreted factor profiles which can alter growth rates/migration of epithelial cells [40]. We did not find a differential effect of conditioned medium from these co-cultures on A549 proliferation. This may have been due to the concentration of growth factors being too low in CM to elicit a proliferative response, however, we cannot rule out the possibility that at the cell-to-cell interphase, the concentration could be locally high enough to elicit a physiological response. In addition, we cannot rule out the possibility, that cell-cell contacts or local integrin activated mechanisms play a role in the observed effect on epithelial wound closure. We have found that IPF co-cultures promote while NHLF co-cultures inhibit re-epithelialization and mesenchymal marker (vimentin) expression in epithelial cells compared to A549 mono-cultures. The inhibitory effect of NHLFs on mesenchymal marker expression in epithelial cells could be detected as early as 24 h whereas the mechanical barrier was sufficiently formed only after 48 h suggesting that soluble factors may be primarily responsible for preventing mesenchymal marker expression in epithelial cells.

PDGF-AA, bFGF, and TGF-β1 have been implicated in the induction of mesenchymal marker expression in epithelial cells [41-43] while HGF can prevent it [25]. NHLFs in co-culture increased the level of HGF while IPF fibroblasts afforded no significant elevation in HGF but a marked increase in bFGF and PDGF-AA suggesting a mechanism for the effect of the two fibroblast phenotypes on mesenchymal marker expression in epithelial cells.

Induction of mesenchymal markers in epithelial cells has been correlated with more rapid migration and wound closure [44] and vimentin expression was shown to be essential and sufficient for promoting alveolar epithelial wound closure *in vitro*[15]. Epithelial to mesenchymal transformation (EMT) of alveolar epithelial cells has been proposed as a potential contributor to the lung fibroblast population in IPF [45,46]. In our study, however, the number of vimentin expressing cells peaked at 48 h and declined thereafter when wound closure was nearly completed. Furthermore, only a partial loss of e-cadherin occurred in vimentin positive epithelial cells. While our results indicate a transient phenomenon rather than a fully completed EMT, the presence of a large number of cells co-expressing epithelial and mesenchymal markers could represent an activated population proactively involved in pro-fibrotic growth factor secretion.

It is well appreciated that cancer and metastasis are underlying complications in IPF [3,47]. Fibroblast-induced aberrant proliferation and mesenchymal marker expression in transformed epithelial cells is perhaps one mechanism by which there is increased risk of lung cancer in IPF. The fact that IPF cells are a greater source of PDGF-AA but fail to respond to this mediator might support the idea that the IPF mesenchyme ‘feeds’ the tumor.

In order to put the unexpected finding of reduced IPF fibroblast proliferation and migration in our co-culture model in a disease context, one could entertain the following hypothesis. In spite of excessive growth factor release following alveolar epithelial cell injury, non-responsive IPF fibroblasts fail to recruit to the wound site leading to insufficient wound contraction and possibly basement membrane damage. Sub-optimal HGF concentrations impair repair and possibly further compensatory increase in PDGF and bFGF concentration may lead to bFGF mediated proliferation and ECM secretion by synthetic, non-migratory IPF fibroblasts and the recruitment and activation of heterogenous fibroblast populations form other areas of the lung forming an activated myofibroblast pool at the injury site.

## Conclusion

Our results described here provide new evidence that the altered phenotype of the IPF fibroblast may play an important role in fibrogenesis and offer new insight into some of the potential mechanisms. Using a novel co-culture model of epithelial injury-repair we demonstrate that the IPF fibroblasts have an aberrant response to epithelial injury. Our data show that the abnormal epithelial-fibroblast cross talk results in the generation of a pro-fibrotic environment and impaired alveolar repair.

## Methods

### Cell culture

A549 cells were obtained from ATCC, primary human lung fibroblasts (NHLFs) from Promocell (Heidelberg, Germany). IPF fibroblasts were obtained from surgical lung biopsies of patients and cultured as described previously [48]. All cell types were maintained in DMEM/F12 medium supplemented with 10% FBS, 1% sodium pyruvate, and 1% antibiotics (Invitrogen, Paisley, UK) at 37°C with 5% CO_2_. Primary fibroblasts were used between passages 4 to 7.

### Co-culture scratch wound assay

Trans-well inserts 24 mm in size with 8 μm pore size (VWR, Leicestershire, UK) were coated with collagen-I at on both sides. A total of 100,000 NHLFs or IPF fibroblasts were allowed to attach to inverted trans-wells for 2 to 3 h. Trans-wells were placed in six well plates with 3 mL complete medium in the lower chamber. The upper chamber of each trans-well was incubated with 2 mL of complete medium containing 500,000 A549 cells or medium only (in case of fibroblast only controls) for a further 4 to 5 h. A549 cells were labeled with the fluorescent dye PKH26 (Sigma). The medium in both chambers was replaced with starvation medium containing 0.05% FBS for 16 h. Three 0.4-0.5 mm parallel scratches were made in the A549 cell layer using a sterile pipette tip. Cells were washed and medium was replaced. For PDGF-AA, PDGF-BB, and Pan-PDGF neutralization, medium was supplemented with 0.5 μg/mL of neutralizing antibody or isotype control (R & D systems).

Experiments were carried out in triplicates with NHLFs (n = 2-4) and IPF fibroblasts (n = 3-5). CM was stored at -80°C for up to 3 days before a single thaw and further analysis and trans-wells fixed with 5% neutral buffered formalin solution (Sigma) for immune-fluorescent staining.

### Immunofluorescent staining

The bottom side of trans-well membranes was wiped clean using moist cotton buds to ensure that only cells on the epithelial side remained. Staining was carried out by incubating with primary antibodies: rabbit anti-e-cadherin (Abcam, Cambridge, UK); mouse anti-α-SMA (Sigma); or mouse anti-vimentin (Dako, Ely, UK) followed by incubation with DAPI/AlexaFluor anti-rabbit488 or anti-mouse647 secondary antibodies (Invitrogen). Stained trans-wells were analyzed using LEICA, TCS SP2 confocal microscope (Leica, Heerbrugg, Switzerland).

### Fibroblast migration assay

After seeding fibroblast as described earlier followed by the 16-h starvation, medium in the top chamber was replaced with 2 mL starvation medium containing 20 ng/mL PDGF-AA or 20 ng/mL bFGF (R & D Systems) for directional migration or starvation medium for non-directional baseline migration.

### Microscopy, image capture, and analysis

Wound closure was monitored in real time by visualizing the fluorescently labeled A549 cells using an inverted microscope (Olympus IX71) equipped with a Nikon camera. Digitized images of three representative areas for every trans-well were captured at 0 h, 24 h, 48 h, 72 h, and 96 h. Images were analyzed using Image-J software (Image-Pro Plus, Version 6.3, Datacell).

To analyze immuno-fluorescently stained trans-wells, 40× images were taken at 10 random points along a centrally placed scratch. At time points where the field of view could not encompass both wound edges lining the denuded area (0 h and 24 h), images were taken to include one wound edge and the denuded area ensuring similar coverage of scratch for all trans-wells. The number of positive staining cells was counted and averaged for all fields. Data were expressed as the number of cells per High Power Field (HPF). Fibroblasts were identified as elongated cells expressing DAPI/α-SMA or DAPI/vimentin. Epithelial cells (A549) were identified as cells expressing E-cadherin/DAPI. A549 cells transiently expressing mesenchymal markers were identified by the presence of triple staining for e-cadherin/vimentin/DAPI.

### Growth factor profiling

CM was analyzed for growth factors. bFGF, HGF, and TGFβ-1 were assessed by using single-plex assays from Meso Scale Discovery (Rockville, MD, USA). Levels of (EGF), IGF-1, PDGF-AA, and PDGF-BB were assessed using Quantikine ELISA (R & D Systems, Abingdon, UK). All assays were performed as per manufacturer’s protocol. For ELISA, CM from four biological replicates were pooled and concentrated 10 times using Amicon Centrifugal Filtration Units (Millipore, Watford, UK). Growth factor concentrations shown in this work fell within the dynamic range of the assays used.

### Cell proliferation assay

A549 cells were plated at 5,000 cells per well in a 96-well assay plate, starved for 48 h, and then stimulated for a further 48 h with CM. Proliferation of fibroblasts in response to PDGF-AA (150 ng/mL) and bFGF (2.4 ng/mL) was assessed in a similar way. Proliferation was assessed using the DELFIA Assay System (Perkin Elmer, Cambridge, UK).

### A549 scratch assay

A549 cells were allowed to reach 100% confluence followed by 24 h starvation. Scratch wounds of 0.3 to 0.4 mm were generated across the wells that were then replenished with either starvation medium or starvation medium containing PDGF-AA (20 ng/mL), bFGF (10 ng/mL). Images were acquired and analyzed as described earlier.

### Immunoblotting

Cells were lysed using RIPA buffer (20 mM Tris-HCl -pH 7.5, 150 mM NaCl, 1 mM EDTA). Proteins were quantified using the Pearce BCA assay kit (VWR). After separation on NUPAGE gels, proteins were transferred to a PVDF membrane and probed overnight with primary antibodies against PDGFRα, PDGFRβ, FGFR1, and GAPDH at 4°C followed by appropriate secondary antibody incubation for 1 h at room temperature. All antibodies were purchased from Cell Signaling Technologies (Boston, MA, USA).

### Statistical analysis

Results were expressed as means ± SEM. Statistical analysis was performed using a one-way ANOVA followed by Dunnett’s post-test. Values of *P* <0.05 were considered statistically significant.

## Abbreviations

bFGF: Basic fibroblast growth factor; CM: Conditioned medium; ECM: Extracellular matrix; EMT: Epithelial mesenchymal transformation; FBS: Fetal bovine serum; FGFR: Fibroblast growth factor receptor; GAPDH: Glyceraldehyde 3-phosphate dehydrogenase; HGF: Hepatocyte growth factor; IL: Interleukin; MCP: Monocyte chemotactic protein; NHLF: Normal human lung fibroblast; PDGF: Platelet-derived growth factor; PDGFR: Platelet-derived growth factor receptor; SMA: Smooth muscle actin; TGF: Transforming growth factor.

## Competing interests

The authors declare that they have no competing interests.

## Authors’ contributions

SP carried out experimental work, contributed to experimental design, data analyses and interpretation, and to the writing of the manuscript. CMH contributed to data interpretation and the writing of the manuscript. GJ contributed to experimental design, data analysis and interpretation, and to the writing of the manuscript. All authors read and approved the final manuscript.

## Supplementary Material

Additional file 1 **Immunofluorescence staining to determine fibroblast invasion into epithelial wound.** Fibroblasts were identified as α-SMA/DAPI positive cells, A549 were identified as e-cadherin/DAPI positive cells. Co-culture with normal fibroblasts (NHLF-1 and NHLF-2) prevented A549 migration and re-epithelialization and filled the wound area progressively. Fewer IPF fibroblasts (IPF-123A, 147B, and 217B) migrated into scratch wound and these co-cultures exhibited enhanced A549 migration and re-epithelialization. Green: e-cadherin, Red: α-SMA, Blue: DAPI. Click here for file

Additional file 2 **Secreted growth factor profile in CM of fibroblasts in monocultores. (A)** bFGF concentration in conditioned medium 48 h or 72 h after serum starvation. **(B)** HGF concentration 48 h or 72 h. **(C)** PDGF-AA concentration 72 h. **(D)** TGF-β1 concentration 48 h and 72 h. Triplicate experiments with multiple donors. Click here for file

Additional file 3 **The effect of pan-PDGF neutralization on epithelial wound closure and fibroblast invasion. (A)** Immunofluorescence staining of the epithelial side of trans-wells (Green: e-cadherin, Red: vimentin, Blue: DAPI). PDGF neutralization resulted in recruitment of fewer NHLFs into epithelial wounds. **(B)** pan-PDGF neutralization resulted in a significant increase in wound closure in NHLF co-cultures while it prevented wound closure in A549 mono-culture and A549-IPF co-culture. *P <0.05, **P <0.01 compared to IgG controls. Click here for file
