# Supplementary figures and images for: Deficient repair response of IPF fibroblasts in a co-culture model of epithelial injury and repair
Source: Fibrogenesis Tissue Repair. 2014 Apr 29;7:7. doi: 10.1186/1755-1536-7-7 (PMC4021590; doi:10.1186/1755-1536-7-7)

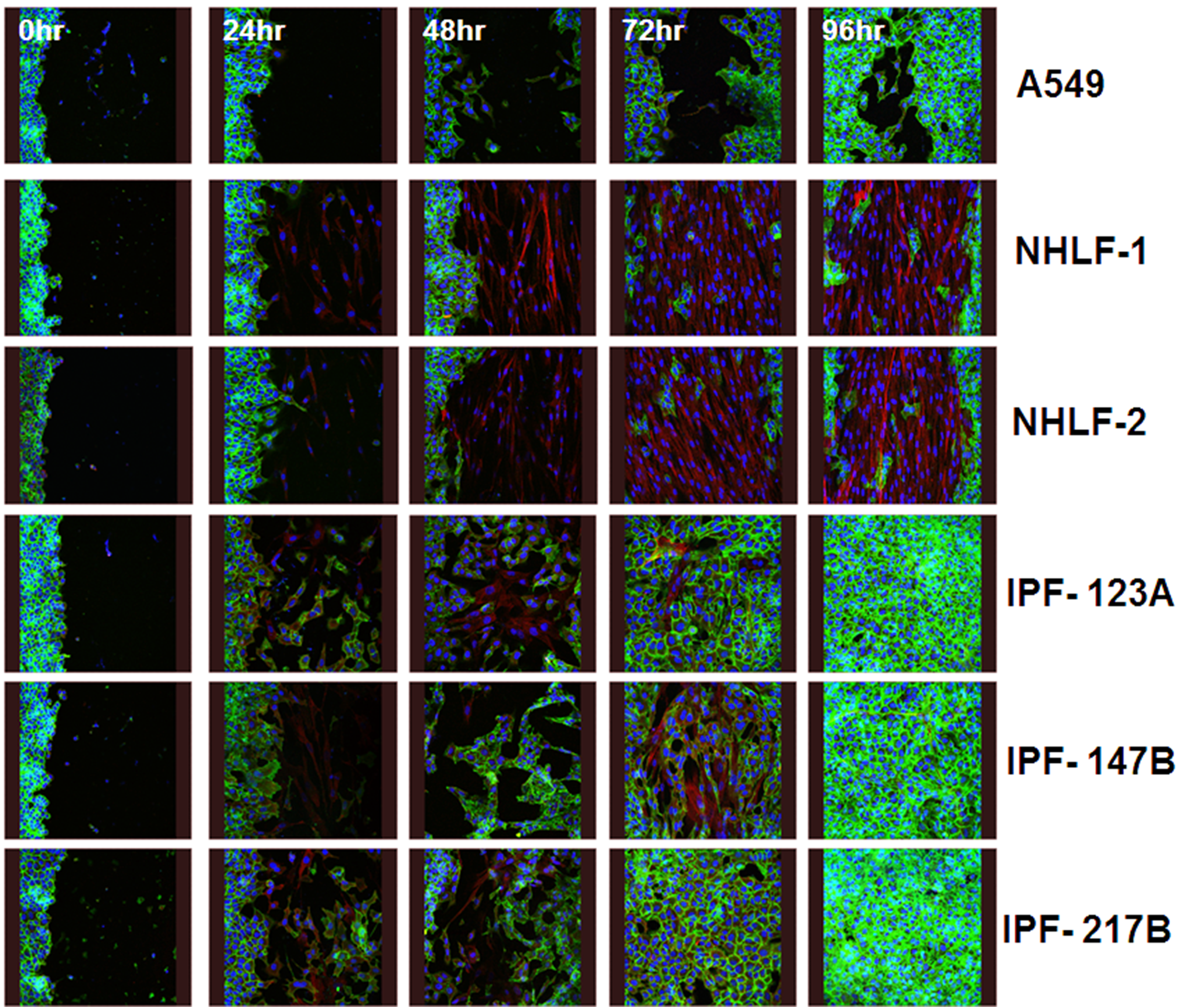

Supplement: Additional file 1 — Immunofluorescence staining to determine fibroblast invasion into epithelial wound. Fibroblasts were identified as α-SMA/DAPI positive cells, A549 were identified as e-cadherin/DAPI positive cells. Co-culture with normal fibroblasts (NHLF-1 and NHLF-2) prevented A549 migration and re-epithelialization and filled the wound area progressively. Fewer IPF fibroblasts (IPF-123A, 147B, and 217B) migrated into scratch wound and these co-cultures exhibited enhanced A549 migration and re-epithelialization. Green: e-cadherin, Red: α-SMA, Blue: DAPI. [file 1755-1536-7-7-S1.tiff]

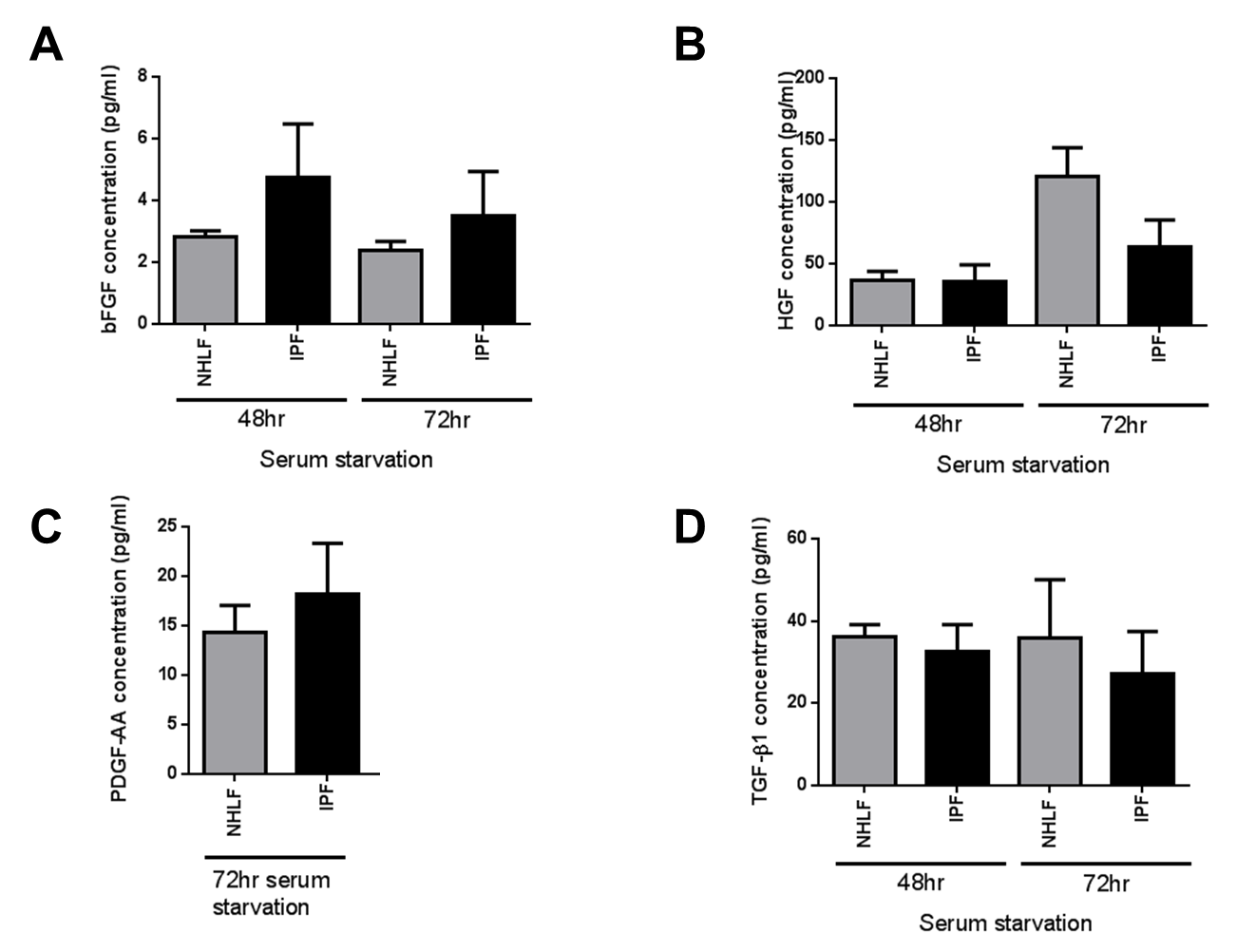

Supplement: Additional file 2 — Secreted growth factor profile in CM of fibroblasts in monocultores. (A) bFGF concentration in conditioned medium 48 h or 72 h after serum starvation. (B) HGF concentration 48 h or 72 h. (C) PDGF-AA concentration 72 h. (D) TGF-β1 concentration 48 h and 72 h. Triplicate experiments with multiple donors. [file 1755-1536-7-7-S2.tiff]

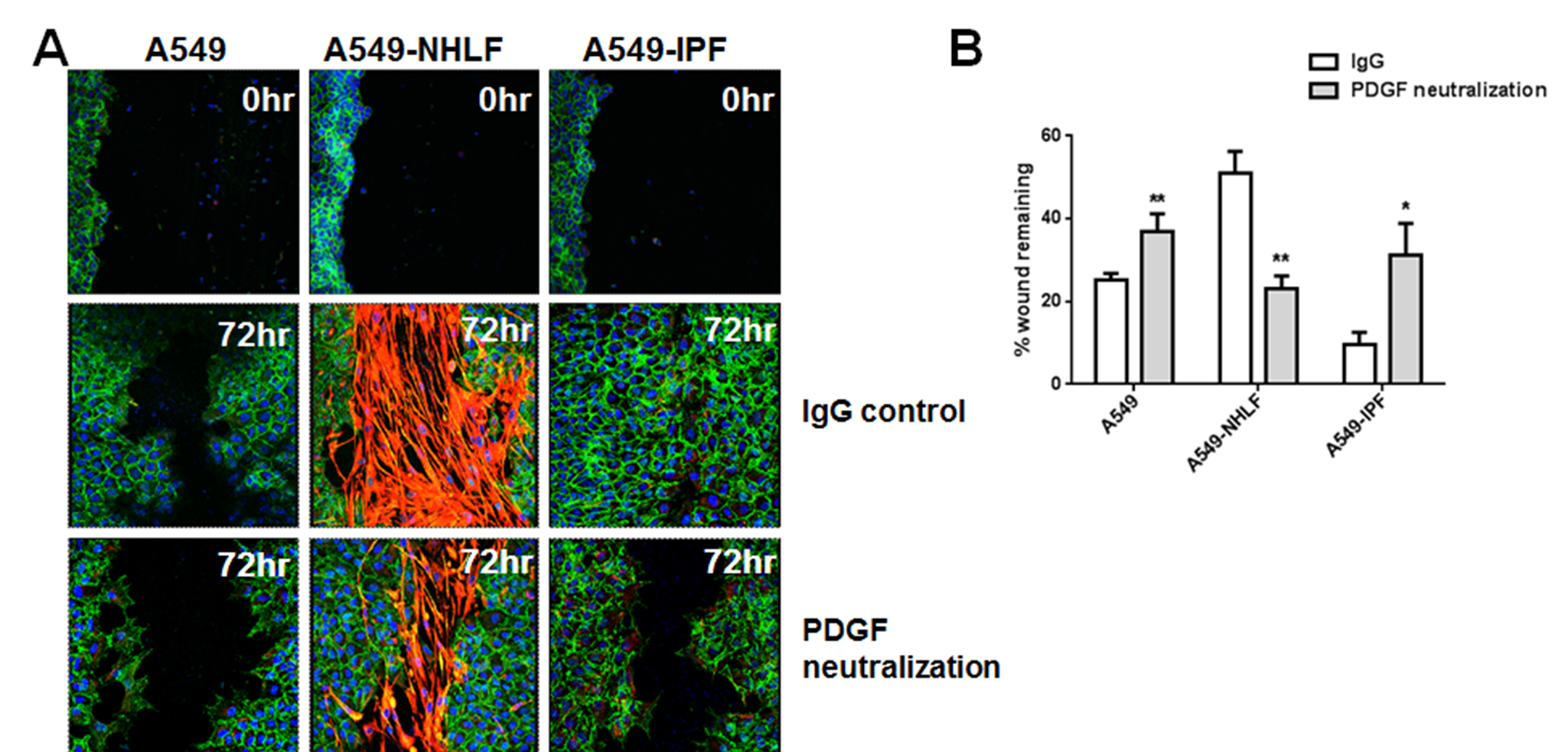

Supplement: Additional file 3 — The effect of pan-PDGF neutralization on epithelial wound closure and fibroblast invasion. (A) Immunofluorescence staining of the epithelial side of trans-wells (Green: e-cadherin, Red: vimentin, Blue: DAPI). PDGF neutralization resulted in recruitment of fewer NHLFs into epithelial wounds. (B) pan-PDGF neutralization resulted in a significant increase in wound closure in NHLF co-cultures while it prevented wound closure in A549 mono-culture and A549-IPF co-culture. *P <0.05, **P <0.01 compared to IgG controls. [file 1755-1536-7-7-S3.tiff]
